# Unique histological features of the tail skin of cotton rat (*Sigmodon hispidus*) related to caudal autotomy

**DOI:** 10.1242/bio.058230

**Published:** 2021-02-19

**Authors:** Marina Hosotani, Teppei Nakamura, Osamu Ichii, Takao Irie, Yuji Sunden, Yaser Hosny Ali Elewa, Takafumi Watanabe, Hiromi Ueda, Takashi Mishima, Yasuhiro Kon

**Affiliations:** 1 Laboratory of Veterinary Anatomy, Department of Veterinary Medicine, School of Veterinary Medicine, Rakuno Gakuen University, Ebetsu, Hokkaido 069-8501, Japan; 2 Laboratory of Anatomy, Department of Basic Veterinary Science, Faculty of Veterinary Medicine, Hokkaido University, Sapporo, Hokkaido, Japan; 3 Department of Biological Safety Research, Chitose Laboratory, Japan Food Research Laboratories, Chitose, Hokkaido, Japan; 4 Laboratory of Agrobiomedical Science, Faculty of Agriculture, Hokkaido University, Sapporo, Hokkaido, Japan; 5 Medical Zoology Group, Department of Infectious Diseases, Hokkaido Institute of Public Health, Sapporo, Hokkaido, Japan; 6 Laboratory of Veterinary Parasitic Diseases, Department of Veterinary Sciences, Faculty of Agriculture, Center for Animal Disease Control, University of Miyazaki, Miyazaki, Japan; 7 Laboratory of Veterinary Pathology, Faculty of Agriculture, Tottori University, Tottori 680-8550, Japan; 8 Department of Histology and Cytology, Faculty of Veterinary Medicine, Zagazig University, Zagazig, Egypt

**Keywords:** Cotton rat, Rat, Caudal autotomy, Tail histology, Collagen

## Abstract

Caudal autotomy in rodents is an evolutionarily acquired phenomenon enabling escape from predators, by discarding the tail skin after traumatic injuries. The histological mechanisms underlying caudal autotomy seem to differ among species. Cotton rats (*Sigmodon hispidus*), which are important laboratory rodents for human infectious diseases, possess a fragile tail. In this study, we compared the tail histology of cotton rats with that of laboratory rats (*Rattus norvegicus*), which have no fragility on their tail, to elucidate the process of rodent caudal autotomy. First, the cotton rats developed a false autotomy characterized by loss of the tail sheath with the caudal vertebrae remaining without tail regeneration. Second, we found the fracture plane was continuous from the interscale of the tail epidermis to the dermis, which was lined with an alignment of E-cadherin^+^ cells. Third, we found an obvious cleavage plane between the dermis and subjacent tissues of the cotton-rat tail, where the subcutis was composed of looser, finer, and fragmented collagen fibers compared with those of the rat. Additionally, the cotton-rat tail was easily torn, with minimum bleeding. The median coccygeal artery of the cotton rat had a thick smooth muscle layer, and its lumen was filled with the peeled intima with fibrin coagulation, which might be associated with reduced bleeding following caudal autotomy. Taken together, we reveal the unique histological features of the tail relating to the caudal autotomy process in the cotton rat, and provide novel insights to help clarify the rodent caudal autotomy mechanism.

## Abstract (precis)

**Summary:** The unique histological structures in derimis, subcutis and coccygeal artery of the tail skin are related to the caudal autotomy mechanism in the cotton rat.

## INTRODUCTION

Autotomy, the phenomenon of discarding or shedding a part of the body, is preserved in some animal species and is generally considered a self-defense mechanism to escape from predators (Emberts et al., 2019). In vertebrates, many lizards are well known as species that can undergo autotomy of the tail, which is followed by subsequent regeneration (Zani, 1996). In mammals, several species of rodents have been reported to possess autotomy of the tail related to predator avoidance as well (Shargal et al., 1999). For example, spiny mice (*Acomys cahirinus*), eastern chipmunks (*Tamias striatus*)*,* and degu *(Octodon degus*) shed their tail skin easily in response to traumatic injuries (Shargal et al., 1999). In contrast with the true autotomy in reptiles, where the entire tail including the caudal vertebra is lost, most instances of autotomy in rodents have been considered a false autotomy, where the tail skin is lost but the vertebra remains (Dubost and Gasc, 1987).

Autotomy in rodents has been reported in five families and at least 27 species, although the precise histology related to the autotomy mechanism varies among species (Shargal et al., 1999). The autotomy process in rodents includes two stages: rupture, and healing of tissue. Specifically for the former, in spiny mice, greater space occupied by hair follicles in the dermis and less absolute connective tissue have been associated with decreased skin elasticity (Seifert et al., 2012). However, the autotomy mechanism has been poorly understood in rodents, except for the spiny mouse, which has been well investigated because of their unique systemic regenerative ability of the injured skin (Shargal et al., 1999; Seifert et al., 2012). In particular, the lack of histological observations on the tail skin in rodents has made it difficult to understand the rupture process of tissue and the variety and/or similarity of mechanisms underlying caudal autotomy among species.

The cotton rat (*Sigmodon hispidus*) is a rodent that is distributed over most of the southern USA. They are classified in the family Cricetidae (with hamsters and gerbils) and belong to a different family from the laboratory rat (*Rattus norvegicus*) and the laboratory mouse (*Mus musculus*), which are classified as Muridae (Coe and Prince, 1996). The cotton rat has been selected as a laboratory animal model in the field of infectious research because it can faithfully reproduce human infectious diseases (Boukhvalova et al., 2009). In addition to its utility in infectious disease research, to date we have reported interesting systemic pathological phenotypes of cotton rats, including chronic kidney disease, chronic anemia, and metabolic disorders (Ichii et al., 2016, 2018, 2020; Nakamura et al., 2019). In addition, the interesting characteristics of cotton rats include a fragile tail (i.e. autotomy); they therefore should not be captured by the tail, as they may spin and shed the tail skin to run away (Faith et al., 1977). Hence, cotton rats can be a useful laboratory animal not only to explore several human diseases but also to investigate the mechanisms underlying the expression of biological characteristics in animals, such as caudal autotomy.

In the present study, we investigated the skin histology of the fragile tail in the cotton rats, especially focusing on the constituent collagen tissue, and compared it with laboratory rats, which do not undergo caudal autotomy. The findings of this study will contribute to further understanding of the autotomy mechanism in rodents and cotton rat biology.

## RESULTS

**Fig. 1.**
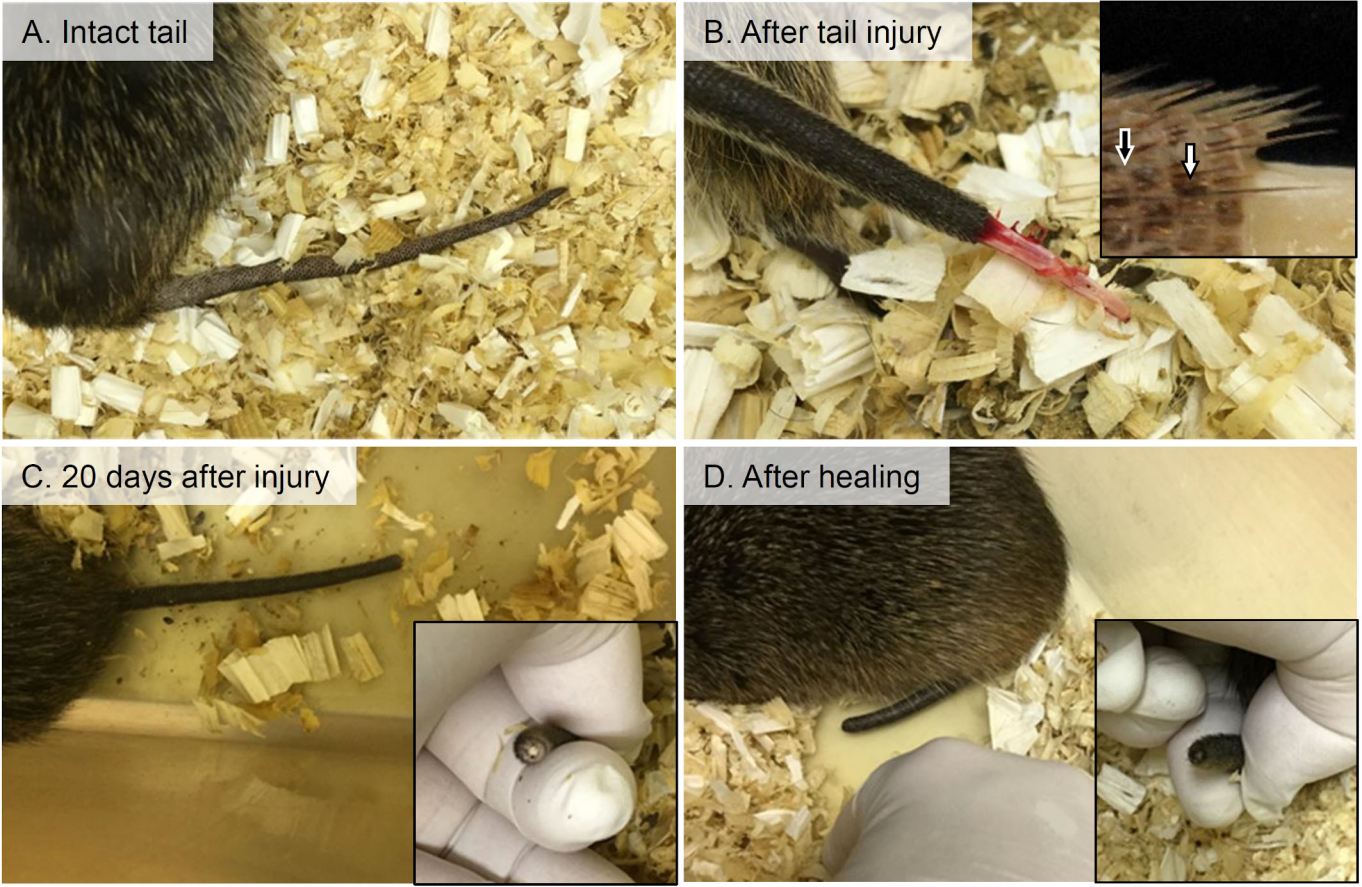
**Macroscopic features of the cotton rat tail.** (A) The intact tail. (B) The tail immediately after injury. Black arrow: interscale; white arrow: scale. (C) The tail at 20 days after injury. (D) The tail after healing.

Fig. 1A shows the intact tail of the cotton rat, while Fig. 1B shows the tail immediately after accidental injury. In the cotton-rat tail, the skin sheath was shed with the caudal vertebrae remaining. The tail epidermis in cotton rats is composed of two distinct regions: the scale and interscale as in other rodents (Gomez et al., 2013), and the tail skin of the cotton rat was ruptured at the latter (Fig. 1B). It is noted that the tail skin of the cotton rat was easily shed without significant bleeding. Although there were large individual differences in the healing of the ruptured tails, the caudal vertebrae were lost at 20 days after the tail injury (Fig. 1C), and the open wound of the injured tail skin was finally covered with new epithelium (Fig. 1D). These results clearly demonstrate that laboratory cotton rats develop false autotomy without tail regeneration and indicate that the tail skin and subcutis possess histological characteristics associated with tail autotomy.

**Fig. 2.**
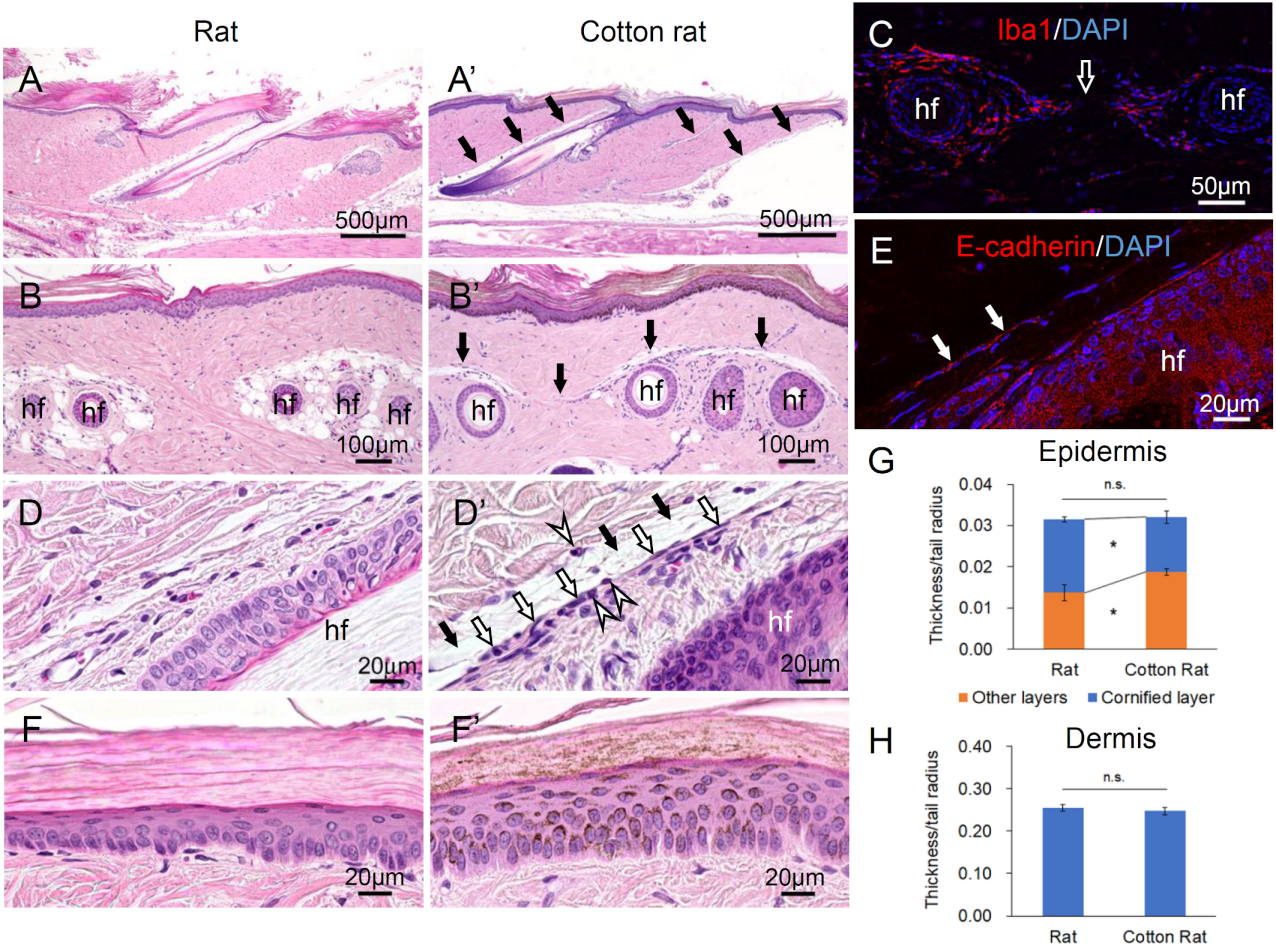
**Histology of tail epidermis and dermis of rat and cotton rat.** (A,A′) Longitudinal section of the tail. (B,B′) Vertical section of the tail dermis. (C) Immunofluorescence of Iba1 as a macrophage marker in cotton-rat tail. (D,D′) Connective tissue around the cranial plane of the hair follicles. (E) Immunofluorescence of E-cadherin in cotton-rat tail. (F,F′) Tail epidermis. (G) Thickness of the epidermis and its constituent layers relative to the tail radius. Significant differences between rat and cotton rat are indicated as *P<0.05 (Mann–Whitney *U*-test). Rat, *n*=6; cotton rat, *n*=10. (H) Thickness of the dermis relative to the tail radius; no significant differences (Mann–Whitney *U*-test). Rat, *n*=6; cotton rat, *n*=10. Black arrows: the crack at the cranial plane of the hair follicles; white arrows: the alignment of cell nuclei; white arrowheads: neutrophils; hf, hair follicle.

Longitudinal histological sections of the tail showed that the tail hair grew in a craniocaudal direction in both rats and cotton rats (Fig. 2A and A′), and that the tail skin of the cotton rat was ruptured at the cranial plane of the hair follicles that was macroscopic at the interscale (Fig. 2A′). The vertical histological sections of the tail showed that the hair follicles in the dermis were arranged in a triplet pair in both rats and cotton rats. In cotton rats, cracks with less connective tissue were observed along the cranial plane of the hair follicles, that is, the fracture plane (Fig. 2A′ and B′). The crack extended over the surface of the next pair of hair follicles and reached the underneath of the epidermis (Fig. 2B′). Iba1^+^ macrophages and neutrophils infiltrated around and between the hair follicles (Fig. 2C and D′). The dermis around the crack was lined with an alignment of flattened cell nuclei in the cotton rat, and these cells expressed E-cadherin (Fig. 2D′ and E). The entire thickness of the tail epidermis did not differ between the rats and cotton rats, whereas the constituent layers significantly differed between the two species: the cornified layer was significantly thinner and the other layers, including the basal, spinous, granular, and clear layers, were significantly thicker in the cotton rats than in the rats (Fig. 2F,F′,G, and H).

**Fig. 3.**
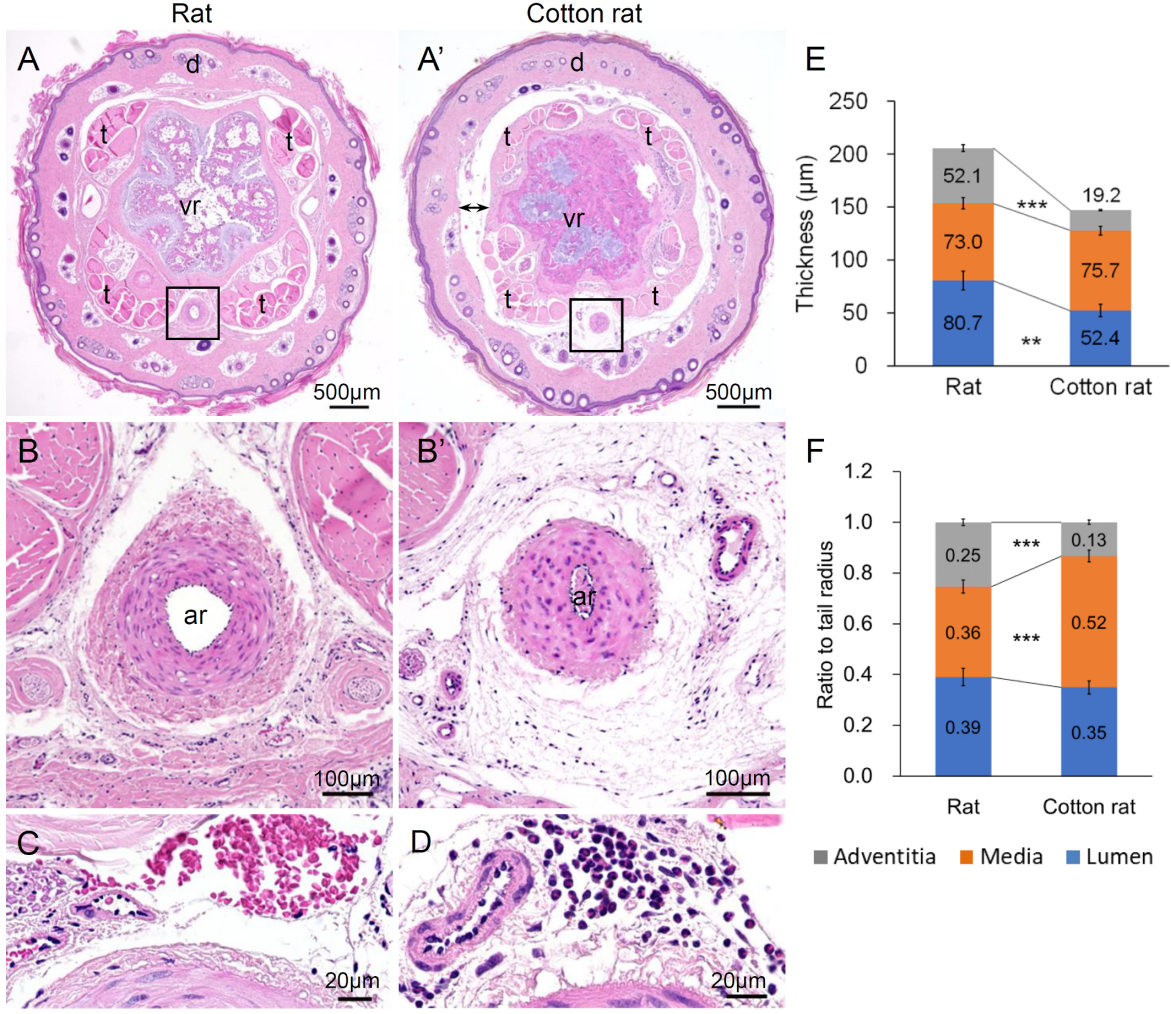
**Histology of tail subcutis of rat and cotton rat.** (A,A′) Cross-section of tail. (B,B′) Tail subcutis and median coccygeal artery. (C) Hemorrhage in the subcutis of cotton rat. (D) Infiltration of neutrophils in the subcutis of cotton rat. (E) Thicknesses of lumen, media and adventitia of the median coccygeal artery. (F) Ratio of lumen, media and adventitia to the radius of the median coccygeal artery. Significant differences between rat and cotton rat are indicated as **P<0.01 and ***P<0.001 (Mann–Whitney *U*-test). Rat, *n*=6; cotton rat, *n*=9. Bidirectional arrow: the cleavage plane between skin and subjacent tissue; ar, the median coccygeal artery; d, dermis; t: tail tendon; vr, caudal vertebrae.

In the cross-sections of the tail, the cleavage plane was observed in the cotton rat between the skin and subjacent tissue, including the tail tendon and muscle (Fig. 3A and A′). The subcutis in the cotton rat was looser than that in the rat (Fig. 3B and B′). Slight hemorrhage and infiltration of neutrophils were observed in the subcutis of the cotton rats (Fig. 3C and D). Regarding the median coccygeal artery, which was the most developed caudal artery in both cotton rats and rats, cotton rats had thinner adventitia than rats, which was confirmed by the statistical analysis of the thickness measurements of these layers (Fig. 3E and F).

**Fig. 4.**
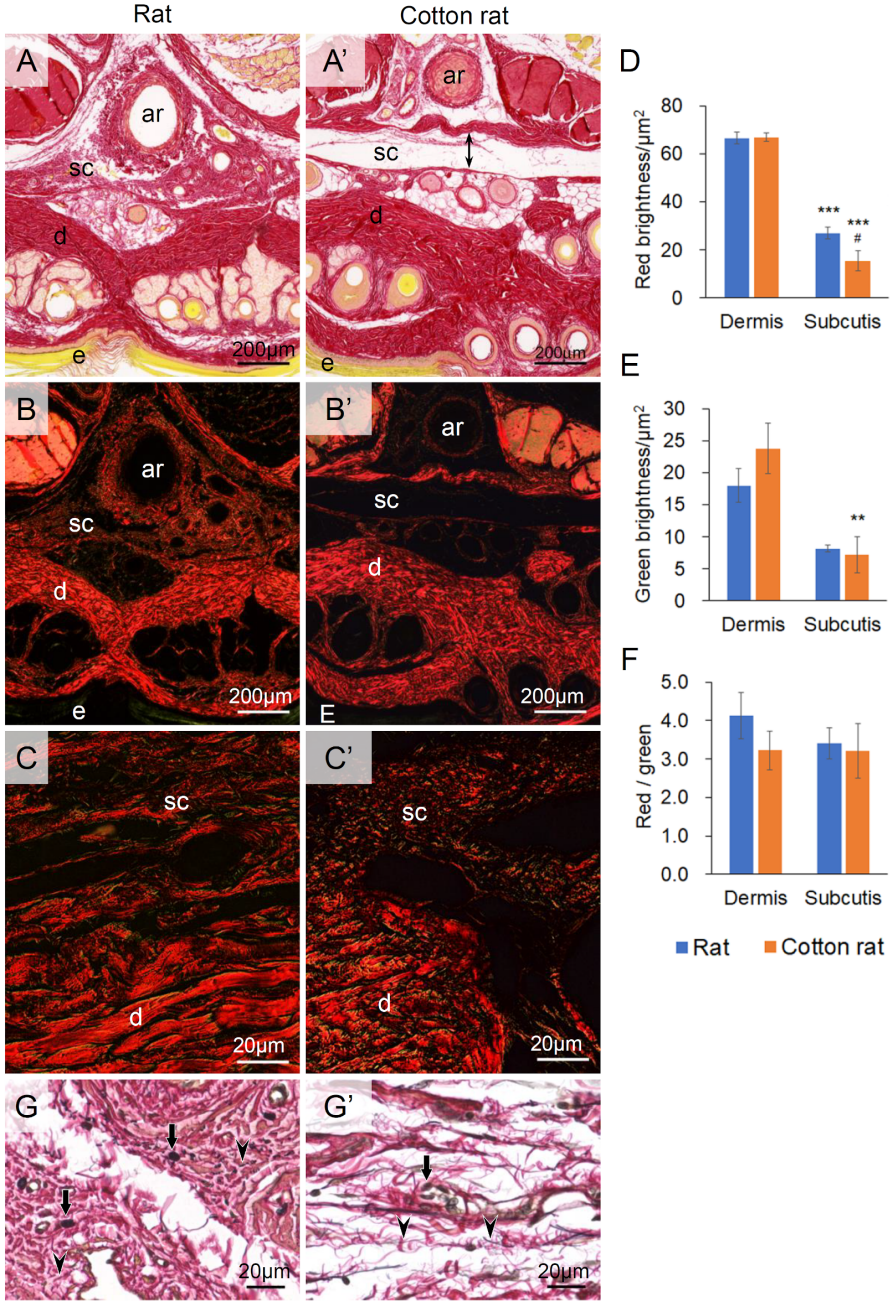
**Distribution of collagen fibers in the tail subcutis of rat and cotton rat.** (A,A′) Tail histology stained by Picrosirius red observed under the light microscope. (B,B′,C,C′) Tail histology stained by Picrosirius red observed under polarized light. Red (D) and green (E) brightness per area in the dermis and subcutis. (F) Brightness ratio of red to green per area in the dermis and subcutis. Significant differences between the dermis and subcutis are indicated as **P<0.01 and ***P<0.001 (Kruskal–Wallis test followed by Scheffé’s method). Significant differences between rat and cotton rat are indicated as #*P*<0.05 (Kruskal–Wallis test followed by Scheffé’s method). Rat, *n*=6; cotton rat, *n*=6. (G,G′) Tail subcutis stained by Elastica van Gieson. Bidirectional arrow: the cleavage plane between skin and subjacent tissue; arrows: bundle elastic fibers; arrowheads: fine elastic fibers; ar: the median coccygeal artery; d, dermis; e, epidermis; sc, subcutis.

Picrosirius red revealed collagen fibers in the dermis, subcutis of the tail skin, and adventitia of the median coccygeal artery as a red color in both rats and cotton rats under a light microscope. The cotton-rat tail showed less distribution of collagen fibers in the subcutis than the rat tail, which corresponds to the cleavage plane in the tail cross-sections (Fig. 4A and A′). Under polarized light, thicker collagen fibrils show strong yellow to red birefringence, while finer collagen fibrils display weak greenish birefringence in the histological sections stained with Picrosirius red (Lattouf et al., 2014). The thicker fibrils shown by red birefringence were more prominent in the dermis than in the subcutis in both rats and cotton rats (Fig. 4B and B′). In the subcutis, the red-stained fibrils were observed as filamentous fibers in rats and as fragmented fibers in cotton rats (Fig. 4C and C′). The red brightness per unit area in the sections was reduced in the subcutis of the cotton rat compared with that of the rat (Fig. 4D). The brightness of green birefringence was also lower in the subcutis than in the dermis in both animal species, and statistically significance was noted in the cotton rat (Fig. 4E). No significant differences were found in the brightness ratio of red to green regardless of animal species and skin layers (Fig. 4F). The elastic fibers composing the subcutis showed a variety of thicknesses in the rat, whereas those in the cotton rat showed fine filamentous morphology (Fig. 4G and G′).

**Fig. 5.**
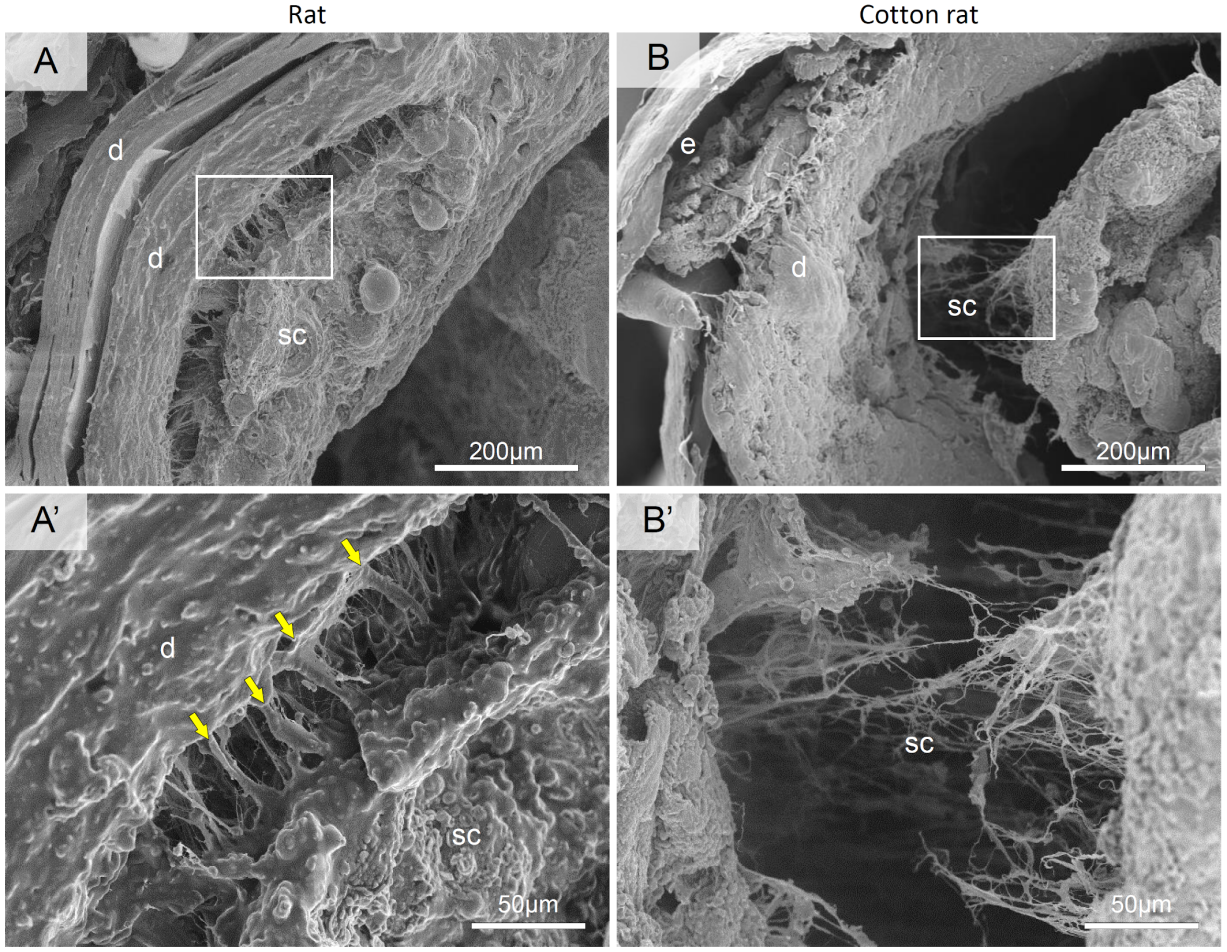
**Ultrastructure of the tail skin layers in rat and cotton rat by scanning electron microscopy.** The images show cross section of the tail skin. The squares in (A,B) are magnified in (A′,B′), respectively. Arrows, fibrous bands. It is noted that the thick fibrous bands connecting between the dermis and subcutis are lacking in the tail of cotton rats.

**Fig. 6.**
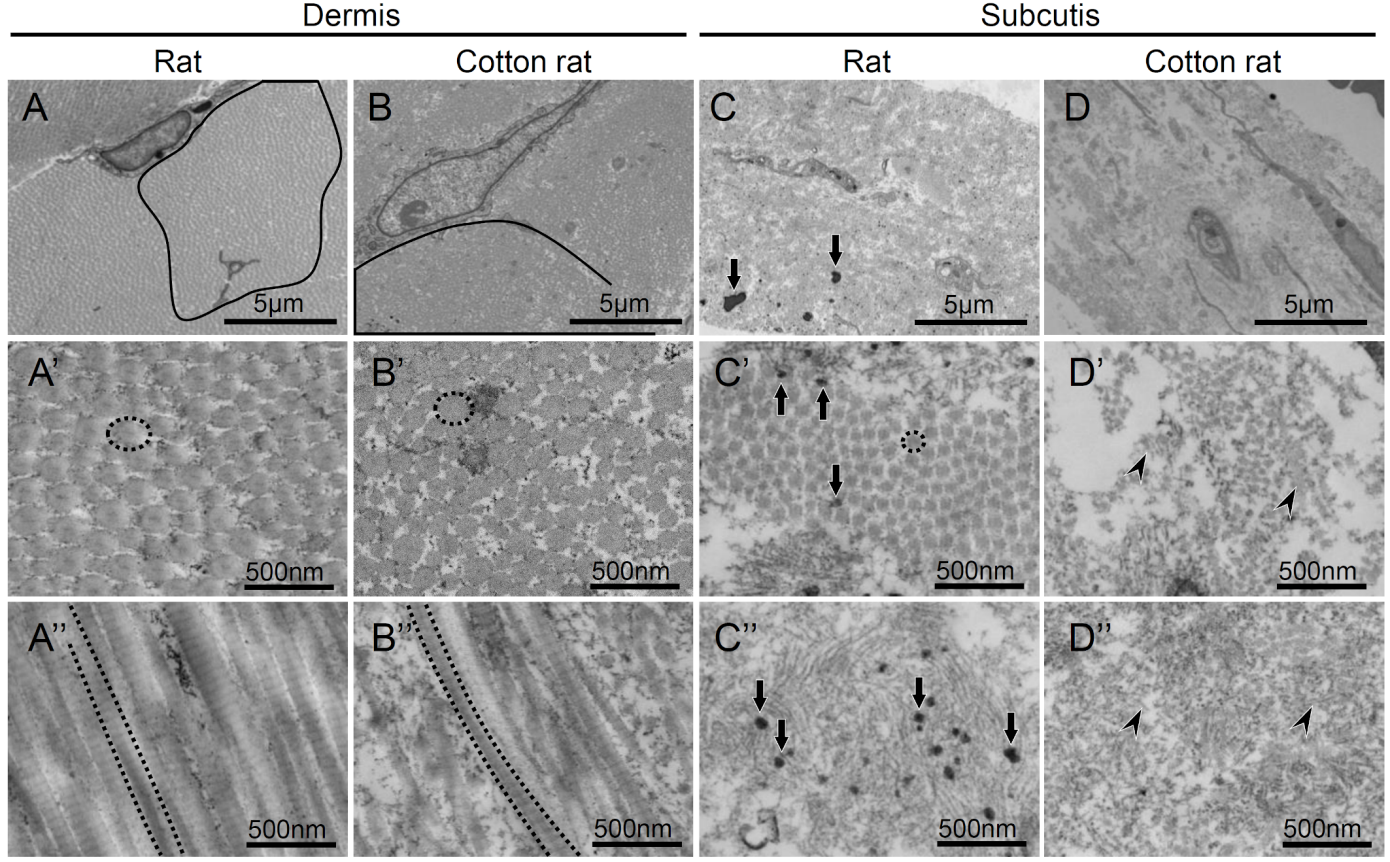
**Ultrastructure of the collagen fibers and fibrils constituting the dermis and subcutis of rat and cotton-rat tails by transmission electron microscopy.** Black lines: collagen fibers; dashed black lines: collagen fibrils; arrows: elastic fibers; arrowheads: fragmented collagen fibrils. Although no apparent differences are found in the tail dermis between species (A-A″,B-B″), fragmented collagen fibrils and fewer elastic fibers are noted in the tail subcutis of the cotton rats (C-C″,D-D″).

The ultrastructural scanning electron microscopic observations of the tail skin layer showed differences between rats and cotton rats (Fig. 5). The subcutis of the rat was attached to the dermis by fibrous bands (Fig. 5A and A′). In the cotton rat, the subcutis underlying the dermis was observed as thin and fine fibers (Fig. 5B and B′). The TEM revealed the differences in the thickness of collagen fibrils composing the skin layers (Fig. 6). Collagen fibers are bundles of densely packed collagen fibrils (Schmitt et al., 1942). Collagen fibrils were observed in the dermis of both rats and cotton rats, with no apparent differences between species (Fig. 6A–A″ and B–B″). In the rat, the subcutis included elastic fibers among the thinner collagen fibrils rather than the dermis, while in the cotton rat, it included fragmented collagen fibrils and finer elastic fibers than the rat (Fig. 6C–C″ and D–D″).

**Fig. 7.**
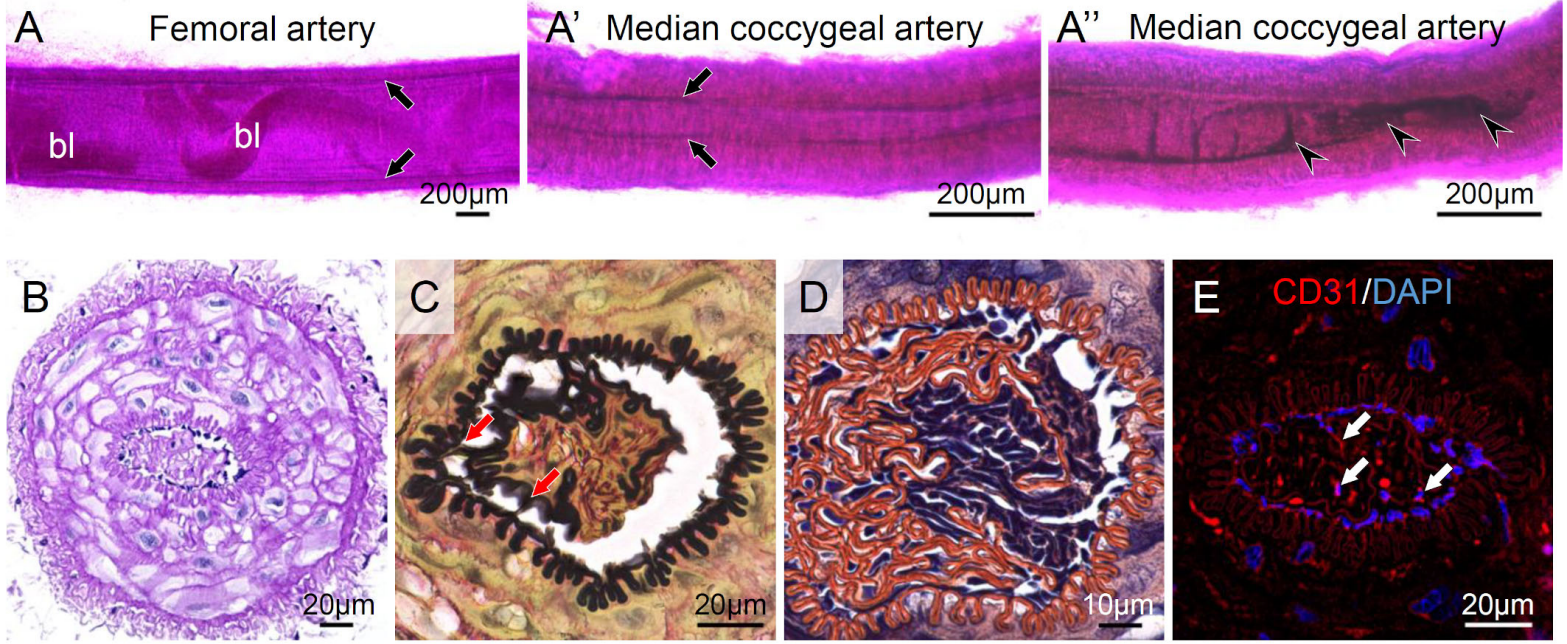
**Histology of the median coccygeal artery of cotton rat.** (A) Whole-mount Periodic acid-Schiff (PAS) staining of the femoral artery of the cotton rat. (A′) Whole-mount PAS staining of the median coccygeal artery with the normal intima. (A″) Whole-mount PAS staining of the median coccygeal artery with the angiostenosis. It is noted that both of the coccygeal tail images were collected from intact tails and the partial angiostenosise along with peeling of the intima were confirmed in all observed cotton rats (*n*=4). Cross-section of the median coccygeal artery stained by (B) PAS, (C) Elastica van Gieson, (D) phosphotungstic acid-hematoxylin. (E) Immunofluorescence of CD31 as an endothelial cell marker. Black arrows: intima; black arrowheads: angiostenosis; red arrows: connection of inner elastic membrane to embolus; white arrows: CD31^+^ endothelial cells in the vascular lumen; bl, blood.

Finally, we report the histology of the median coccygeal artery of the cotton rat. Whole-mount PAS staining showed intima and blood in the lumen of the femoral artery of the cotton rat (Fig. 7A). On the other hand, the median coccygeal artery of the cotton rat showed partial angiostenosis along with peeling of the intima (Fig. 7A′ and A″). The PAS-positive embolus was found in the median coccygeal artery lumen (Fig. 7B), which was continuous with the inner elastic membrane and entangled with fibrin (Fig. 7C and D). The embolus contained CD31^+^ endothelial cells (Fig. 7E).

## DISCUSSION

This study showed that cotton rats develop false caudal autotomy, which involves unique histological features in the skin and subcutis. The first step of the false caudal autotomy is tearing of the epidermis and dermis after pressure or pulling is exerted, whose mechanism differs among species. In cotton rats, the tail skin is ruptured at the designated fracture plane, which is anatomically the interscale of the tail epidermis, and histologically the dermis was lined with E-cadherin^+^ cells at the cracks along the cranial plane of the hair follicles. E-cadherin is an adhesion molecule that plays a role in epithelial cell behavior (van Roy and Berx, 2008). In postnatal mice, E-cadherin^+^ cells appear in the tail dermis, and these cells do not exhibit mesenchymal properties characterized by the expression of fibronectin and vimentin (Kong et al., 2006). In the cotton rat, the dermal fracture plane seemed to be partitioned by E-cadherin^+^ epithelium-prone cells from dermal layers. The loss of connective tissue synthesis in E-cadherin^+^ cells might possibly result in the rupture of the fragile plane in the cotton rat. The histology of the fracture plane varies among the animal species (Shargal et al., 1999). In reptiles, the fracture plane in the tail epidermis is the thinnest cornified layer (Gilbert et al., 2013), and the plane in the tail dermis is characterized by the alignment of cells containing secretory granules, the so-called cellular zipper (Sanggaard et al., 2012; Gilbert et al., 2013). Secretory products from the cellular zipper are likely to be involved in collagen degeneration in the dermal fracture plane (Shargal et al., 1999). On the other hand, the spiny mouse has no fracture planes in the epidermis and dermis of the tail skin, but has larger hair follicles and a smaller area of connective tissue in the dermis, which are related to the fragile skin (Shargal et al., 1999).

The second step of the false caudal autotomy in rodents is to shed the skin sheath, including the epidermis, dermis, and subcutis, whereas the vertebrae remain. The cross-sections of the cotton-rat tail showed an obvious cleavage plane between the dermis and subjacent tissues. Similar to this, the cleavage plane between the subcutis and underlying tissues also exists in the spiny-mouse tail (Shargal et al., 1999). The designated fracture or cleavage plane is estimated to reduce bleeding and subsequent bacterial infection and induce rapid wound healing (Shargal et al., 1999; Maginnis, 2006). Taken together, these results indicate that multiple mechanisms are involved in caudal autotomy in cotton rats, and the anatomical and histological phenotypes of the cotton-rat tail are adaptive to the caudal autotomy.

The ultrastructural differences of the collagen fibers in the skin reflect the differing fragility of the skin. For instance, disruption of the gene locus coding *decorin* in mice results in a looser network of collagen fibers with a randomized diameter of collagen fibrils, so that the mutant mice show the phenotype of skin fragility and easy tail shedding (Danielson et al., 1997). In humans, the loss and fragmentation of dermal collagen fibrils lead to weakened and fragile skin (Quan and Fisher, 2015). In the dermis of cotton rats, the abundance of collagen fibers and fibril diameter are comparable to those of the rat, as shown by Picrosirius red staining and TEM. Therefore, tail rupture in the dermis of the cotton rat does not result from differences in the properties of collagen fibers, but from the aforementioned structural characteristics of the fracture plane. On the other hand, Picrosirius red staining showed lower abundance of the collagen fibers in the subcutis of the cotton rat. In addition, the subcutis in the cotton rat was composed of thinner fine collagen fibrils and less elastic fibers than in the rat. Therefore, thinner collagen fibrils might decrease the amount of collagen fibers in the subcutis of cotton rats and correspond to the cleavage plane between the skin and subjacent tissue under the light microscope. Generally, the fibrous bands in the subcutis play a role in connecting the dermis to the hypodermis and the skin to the deep fascia (Jacobs et al., 2018). However, the subcutis of the cotton rat cannot keep a tight connection of the skin sheath to the tail when an external force is received because of its loose network of collagen and elastic fibers, suggesting the fragility of the connective tissues in the subcutis facilitates easy autotomy of the tail skin in the cotton rat. Collagen and elastin molecules are largely synthesized by connective tissue cells, including fibroblasts, which are regulated by interactions in the extracellular matrix among growth factors, hormones, and cytokines (Pawlina and Ross, 2018). Further studies are needed to clarify the molecular mechanism resulting in compartmental differences in the extracellular matrix environment and the property of collagen fiber production in the skin of cotton rats.

Immune cells such as neutrophils and macrophages infiltrate along the fracture plane and in the subcutis. These immune cells are rapidly recruited to the injured tissue by danger-associated molecular patterns (Zhang and Mosser, 2008; Kovtun et al., 2018). It is likely that the loose attachment induces persistent friction of the fracture plane, recruiting innate immunity. Furthermore, the resident innate immune cells might contribute to the quick defence against the invasion of exogenous pathogens from the wound, which would be one of the autotomy adaptations in rodents.

After shedding the tail skin, the cotton rats showed less bleeding. The median coccygeal artery of the cotton rat possesses a thick smooth muscle layer, and its intima and interelastic membrane were partially peeled into the vascular lumen with fibrin entanglement. Incomplete angiostenosis by these unique structures is considered to be a mechanism to reduce bleeding following caudal autotomy in the cotton rat. Many reptiles have anatomical characteristics on their vasculature to minimize bleeding after the autotomy; the constriction of sphincter smooth muscles on the caudal artery and valves in the caudal vein anterior to the injured point (McLean and Vickaryous, 2011; Stebbins and McGinnis, 2012; Gilbert et al., 2013). This study is the first report indicating the caudal vasculature adaptation of the rodent to autotomy. The hemorrhage in the subcutis of the cotton rat indicates the fragility of blood vessels, including the arteriole, because of the thin adventitia and the exposure of a continuous external force to blood vessels by the loose connection of the subcutaneous tissue. Mechanical stress has been reported to accelerate the cellular proliferation of the aortic smooth muscle (Kawaguchi et al., 2001). The three layers of blood vessels interact with each other; the adventitia mediates communication between intimal endothelial cells and medial smooth muscle cells, and their neighboring environment (Majesky et al., 2011). This study could not define the causes inducing the peeling of intima; however, the constant external stress on the vessels and altered local environment in the subcutis might enhance intimal hyperplasia.

Autotomy in animals helps them to escape from predators and increases survival and reproductive success, and it has been reported that predation risk might progress the evolution of autotomy (Lin et al., 2017). The lifespan of wild cotton rats is less than 6 months (Cameron and McClure, 1988). On the other hand, the laboratory cotton rat survives up to 23 months (Faith et al., 1977; Curlee and Cooper, 2012). Although the cause of the shorter lifespan in wild cotton rats is still not fully elucidated, predation by carnivorous birds and mammals (Morris and Conner, 2019) and high susceptibility to pathogenic viruses (Ehlen et al., 2016) might cause a shorter lifespan in the wild than in the laboratory. It is likely that the cotton rat acquired caudal autotomy during its evolution to minimize the loss of individuals, as reported in other rodents performing autotomy (Shargal et al., 1999).

In conclusion, caudal autotomy mechanism of the cotton rat includes multiple unique histological structures: the designated fracture plane lined with E-cadherin^+^ cells in the dermis, fine collagen fibers, and less elastic fibers composing the subcutis, and histopathological changes of the median coccygeal artery adapted to the easy injury. These findings provide new information to explain autotomy and the variety of its mechanisms in rodents.

## MATERIALS AND METHODS

### Animals

Animal experimentation was performed in accordance with the guidelines issued by the Hokkaido Institute of Public Health (approval no. K27-03 and K30-01), and by Chitose Laboratory, Japan Food Research Laboratories (approval no. HK190308-02). Inbred cotton rats (*Sigmodon hispidus*) maintained at Hokkaido Institute of Public Health (HIS/Hiph), and outbred laboratory rats (JclBrlHan:WIST) purchased from CLEA Japan (Tokyo, Japan) were used in the present study. Male cotton rats with the intact tail at adult age (6 to 10 months, *n*=11) and body-weight-matched male laboratory rats at approximately 8 weeks of age (*n*=6) were used. If age-matched cotton rats were used at 8 weeks of age, the body weight and the tail diameter of the cotton rats were twofold smaller than those of the rats (data not shown), which makes it difficult to compare the tail histology between these animals. Together with the fact that rats do not show the phenotype of caudal autotomy regardless of their age, the body-size- and tail-diameter-matched comparison seems to be suitable for examining the structural differences between cotton rats and rats morphologically and histologically.

The animals were euthanized by cutting the abdominal aorta under deep anesthesia using isoflurane, and their tails were collected. The tails of cotton rats with intact tails and rats were subjected to histological and ultrastructural experiments. For the examination of the fracture plane in Figs 1B and 2A′, a total of four cotton rats were either stripped of their tail under deep anesthesia (*n*=3) or euthanized shortly after the tail rupture (*n*=1).

### Light microscopy

The tails were fixed with 10% neutral buffered formalin, decalcified using Morse's solution (10% sodium citrate and 20% formic acid, Fujifilm Wako, Osaka, Japan), and embedded in paraffin. The sliced sections were stained with hematoxylin and eosin (HE), Picrosirius red, Elastica van Gieson, periodic acid Schiff (PAS), and phosphate tungsten acid hematoxylin (rat, *n*=6; cotton rat, *n*=9). The coccygeal arteries were removed from the fixed intact tail, cut into approximately 1 cm, and stained with whole-mount PAS. Specifically, the arteries were immersed in 0.5% periodic acid solution for 5 min, stained with Schiff reagent for 5 min, and washed with sulfurous acid solution for 10 min followed by tap water. The stained arteries were immersed in graded glycerol for the penetration and removal of excess staining. The sections were observed using an all-in-one fluorescence microscope (BZ-X800, Keyence, Osaka, Japan). For Picrosirius red staining, the sections were also observed using a microscope attached with polarized filters (Keyence) (rat, *n*=6; cotton rat, *n*=6).

### Immunofluorescence

The sections were deparaffinized, heated with 0.01 M sodium citrate buffer (pH6.0; LSI Medience, Tokyo, Japan) for 40 min at 95°C, and incubated with 2% bovine serum albumin for 30 min, followed by rabbit anti-Iba1 polyclonal antibody (1:3000, Fujifilm Wako), rabbit anti-E-cadherin antibody (1:100, Santa Cruz, TX, USA), and rabbit anti-CD31 polyclonal antibody (1:100, Abcam, Cambridge, UK) overnight at approximately 4°C. Next, the sections were treated with Alexa Fluor 594 donkey anti-rabbit IgG antibodies (1:1000; Life Technologies, Carlsbad, CA, USA) for 30 min at approximately 25°C, and coverslipped with DAPI Fluoromount-G (SouthernBiotech, AL, USA). The sections were observed using an all-in-one fluorescence microscope (BZ-X800, Keyence) (cotton rat, *n*=4).

### Scanning electron microscopy

The tails were removed and fixed using a half-strength Karnovsky's fixative solution [2.5% glutaraldehyde, 2% paraformaldehyde, 0.1 M phosphate buffer (PB), pH 7.4] (rat, *n*=3; cotton rat, *n*=3). After six washes in 0.1 M PB, these organs were post-fixed with 1% osmium tetroxide in 0.1 M PB for 2 h at room temperature. After six washes in distilled water, the specimens were subjected to conductive treatment with 5% BEL-1 (Nisshin EM Co. Ltd., Tokyo, Japan) in 70% ethanol for 2 h at room temperature. Specimens were dried completely and sputter-coated with gold using an ion-sputter E102 (Hitachi, Tokyo, Japan). Specimens were examined using an S-2460N scanning electron microscope (Hitachi, Tokyo, Japan).

### Transmission electron microscopy

The tails were removed and fixed using a half-strength Karnovsky's fixative solution (rat, *n*=3; cotton rat, *n*=3). The tails containing bone tissue were decalcified by immersion in 4.13% EDTA for 10 days. The contrast of fibrotic structures was enhanced for transmission electron microscopy by heavy metal block staining, as described previously (Thai et al., 2016). Briefly, the fixed specimens were washed four times with a solution containing 0.1 M cacodylate buffer (pH 7.4) and 2% OsO_4_ (TAAB Laboratories Equipment Ltd., Berkshire, UK) in 0.15% K_4_(CN)_6_ (Nacalai Tesque, Inc., Kyoto, Japan) for 4 min each at 4°C and soaked in the same solution for 1 h. After four washes with distilled water (4 min each), the samples were immersed in 0.1% thiocarbohydrazide (Sigma-Aldrich Co. LLC., St. Louis, MO, USA) for 20 min at room temperature, washed again with distilled water, and immersed in 2% OsO_4_ for 30 min at room temperature. After further washing with distilled water, the samples were immersed in 1% uranyl acetate at 4°C overnight, washed again in distilled water, and immersed in Walton's lead aspartate solution at 60°C for 30 min. Finally, the samples were dehydrated through an ethanol series, transferred to QY-1, and embedded in epoxy resin (Quetol 812; Nissin EM Co. Ltd., Tokyo, Japan). The tissue block was sliced into 80 nm thick sections perpendicular to the long axis of the myofiber using an ultramicrotome (JUM-7; JEOL Ltd., Tokyo, Japan). The sections were collected on a 100 mesh copper grid and the structures were observed using transmission electron microscope (HT-7700; Hitachi High Technology Co., Tokyo, Japan) at an acceleration voltage of 80 kV.

### Statistical analysis

Results were expressed as means±standard error of the mean (s.e.m.) and statistically analyzed in a non-parametric manner. Data between the two groups were compared using the Mann–Whitney *U*-test. The Kruskal–Wallis test was used to compare three or more groups. When significant differences were observed (*P*<0.05), multiple comparisons were performed using Scheffé’s method.
